# Seneca Valley Virus 3C^pro^ Cleaves PABPC1 to Promote Viral Replication

**DOI:** 10.3390/pathogens9060443

**Published:** 2020-06-04

**Authors:** Qiao Xue, Huisheng Liu, Zixiang Zhu, Zhaoning Xue, Xiangtao Liu, Haixue Zheng

**Affiliations:** State Key Laboratory of Veterinary Etiological Biology, National Foot and Mouth Diseases Reference Laboratory, Key Laboratory of Animal Virology of Ministry of Agriculture, Lanzhou Veterinary Research Institute, Chinese Academy of Agricultural Sciences, Lanzhou 730046, China; xueqiao@caas.cn (Q.X.); liuhuisheng@caas.cn (H.L.); zhuzixiang@caas.cn (Z.Z.); xuezhaoning@yeah.net (Z.X.); liuxiangtao@caas.cn (X.L.)

**Keywords:** seneca valley virus, 3C^pro^, antagonistic mechanism, PABPC1, translation

## Abstract

Seneca Valley Virus (SVV) is an oncolytic virus of the Picornaviridae family, which has emerged in recent years. The impact of SVV on host cell translation remains unknown. Here, we showed, for the first time, that SVV infection cleaved poly(A) binding protein cytoplasmic 1 (PABPC1). In SVV-infected cells, 50 kDa of the N terminal cleaved band and 25 kDa of the C terminal cleaved band of PABPC1 were detected. Further study showed that the viral protease, 3C^pro^ induced the cleavage of PABPC1 by its protease activity. The SVV strains with inactive point mutants of 3C^pro^ (H48A, C160A or H48A/C160A) can not be rescued by reverse genetics, suggesting that sites 48 and 160 of 3C^pro^ were essential for SVV replication. SVV 3C^pro^ induced the cleavage of PABPC1 at residue 437. A detailed data analysis showed that SVV infection and the overexpression of 3C^pro^ decreased the protein synthesis rates. The protease activity of 3C^pro^ was essential for inhibiting the protein synthesis. Our results also indicated that PABPC1 inhibited SVV replication. These data reveal a novel antagonistic mechanism and pathogenesis mediated by SVV and highlight the importance of 3C^pro^ on SVV replication.

## 1. Introduction

Seneca Valley Virus (SVV) was firstly isolated in the United States (US) in 2002 as a contaminant in the cell culture of human fetal retinoblasts [1,2]. Subsequently, a large number of SVV infections have been reported in the US, Colombia, Thailand, Canada, Brazil, and China, which were associated with porcine idiopathic vesicular disease and caused significant economic consequences [3,4,5,6,7,8]. In China, the first case of SVV infection was observed in Guangdong Province in 2015 [9]. Subsequently, SVV infections were reported on other farms in Guangdong and Hubei Provinces from 2015 to 2016 [10,11]. In 2017, we also isolated novel SVV strains in Fujian and Henan Provinces, China [2].

SVV, a positive single-stranded RNA virus, belongs to the Picornaviridae family. The SVV genome is a 7.3-kb molecule, which encodes a single polyprotein that is subsequently cleaved by viral 2A and 3C protease to produce mature structural proteins and non-structural ones [2,12]. SVV also is an effective oncolytic virus against several human cancers [13,14,15,16]. Similar to other proteins of picornaviruses, SVV 3C^pro^ also possesses a conserved catalytic box with Cys and His residues. In addition, SVV 3C^pro^ inhibits host innate immune responses by the cleavage of mitochondrial antiviral-signaling (MAVS) protein, Toll/IL-1R-domain-containing adaptor-inducing IFN-β (TRIF), and TRAF family member-associated NFκB activator (TANK) and the reduction in interferon regulatory factor (IRF)3/7 protein expression caused by its protease activity [12,17]. Picornaviruses’ 3C proteases have evolved a variety of strategies to modulate the host cell translation process, with the aim of promoting viral mRNA translation and replication [18]. However, the relationship between SVV and host cell translation remains unclear.

It is well known that eukaryotic translation initiation factors (eIFs) play important roles in host protein translation [19,20,21,22]. During this process, eIF4G is the most important member of the eIF4F complex. The poly(A) binding protein cytoplasmic 1 (PABPC1, also known as PABP) promotes translation initiation by interaction with eIF4G and by facilitating the binding of 60S ribosomal subunits to 48S preinitiation complexes at the last step of initiation [21]. PABPC1 is a conserved protein. The amino acid sequences of human and porcine PABPC1 are 100% similar. To date, many viruses have developed strategies that cleave, reduce, or utilize PABPC1 protein to regulate viral replication [23,24,25]. SVV is an emergent virus. The relationship between SVV and PABPC1 protein should be investigated to uncover the pathogenesis of SVV in the infected cells.

In the present study, we evaluated the status of PABPC1 protein during SVV infection. Our results determined that SVV infection induced the cleavage of PABPC1, and 3C protease activity played an important role in this process. In addition, SVV infection and the overexpression of 3C^pro^ inhibited the host proteins’ translation rates. We also demonstated the important antiviral role of PABPC1 in SVV replication. These data suggest novel pathogenic mechanisms of SVV that were mediated by 3C^pro^.

## 2. Results

### 2.1. SVV Infection Cleaved PABPC1 Protein

**Figure 1:**
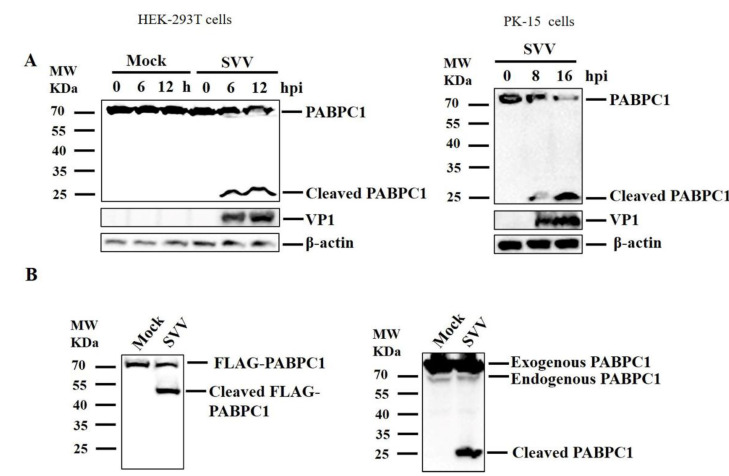
Seneca Valley Virus (SVV) infection cleaved poly(A) binding protein cytoplasmic 1 (PABPC1). (**A**) human embryonic kidney 293T (HEK293T) (left) or porcine kidney 15 (PK-15) cells (right) were cultured in 3.5-cm dishes, and the monolayer cells were mock-infected or infected with SVV (MOI 1). The cells were collected at the indicated time points after infection and analyzed by western blotting. (**B**) HEK293T cells were cultured in 3.5-cm dishes, and the monolayer cells were transfected with 1 μg FLAG–PABPC1 plasmid for 24 h. The cells were mock-infected or infected with SVV (MOI 1) for 12 h. The target proteins were analyzed by western blotting.

To investigate the impact of SVV on PABPC1 protein, human embryonic kidney 293T (HEK293T) or porcine kidney 15 (PK-15) cells were cultured in 3.5-cm dishes, and the monolayer cells were mock-infected or infected with SVV at a multiplicity of infection (MOI) of 1. The cells were collected and analyzed by western blotting. SVV infection induced cleavage of endogenous PABPC1, generating approximately 25 kDa cleaved bands (Figure 1A).

In this study, an anti-PABPC1 polyclonal antibody that detects the carboxyl terminal regions of PABPC1 was used. Therefore, the cleaved bands of PABPC1 induced by SVV should be the carboxyl terminal regions of PABPC1. To detect N terminal regions of PABPC1, HEK293T cells were transfected with N terminal FLAG-tagged PABPC1 plasmid. At 24 h post-transfection (hpt), the cells were infected with SVV (MOI 1) for 12 h. The target proteins were analyzed by western blotting using anti-FLAG and anti-PABPC1 antibodies. This showed that SVV infection also cleaved exogenous PABPC1, generating approximately a 50 kDa of N terminal cleaved band and a 25 kDa of C terminal cleaved band (Figure 1B). Taken together, our results indicate that SVV infection cleaves PABPC1 protein into 50 kDa of the N terminal cleaved band and 25 kDa of the C terminal cleaved band.

### 2.2. SVV 3C^pro^ Cleaved PABPC1, and 3C^pro^ Protease Activity Was Responsible for this Effect

**Figure 2:**
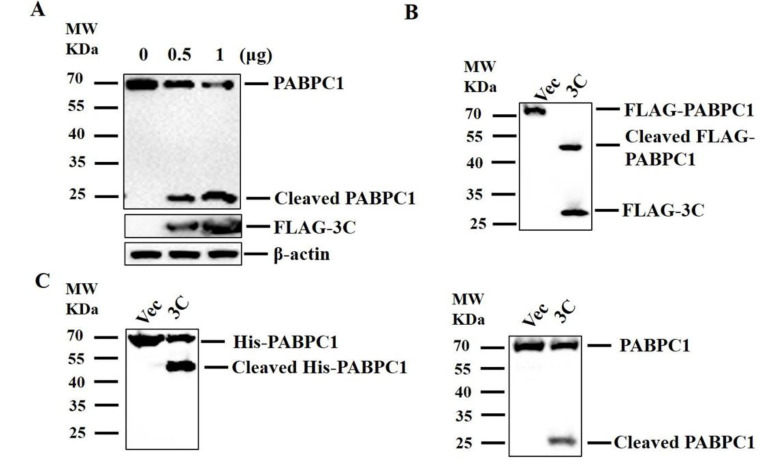

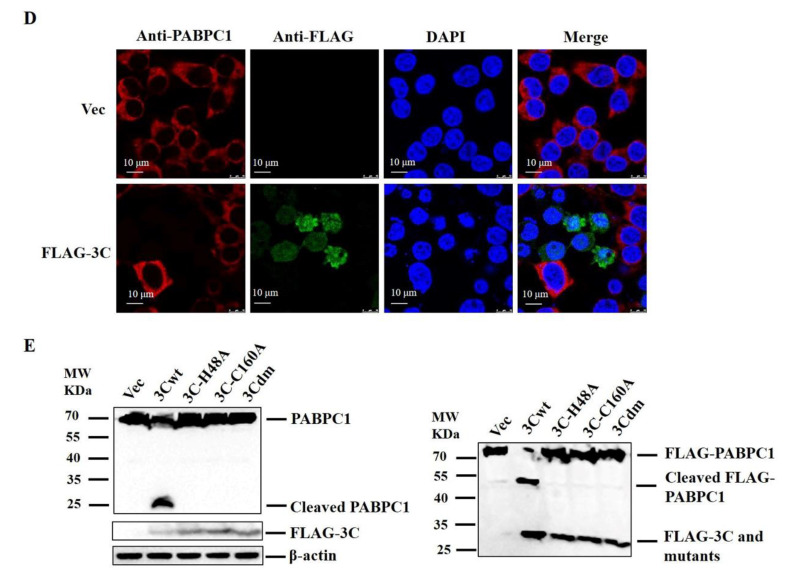
SVV 3C^pro^ cleaved PABPC1, and 3C^pro^ protease activity was essential for this process. (**A**) HEK293T cells were cultured in 3.5-cm dishes, and the monolayer cells were transfected with 0, 0.5, and 1 μg FLAG–3C-expressing plasmids or empty FLAG vectors. At 24 hpt, the cells were collected and analyzed by western blotting. (**B**) HEK293T cells were cultured in 3.5-cm dishes, and the monolayer cells were transfected with 1 μg FLAG–PABPC1 plasmid along with 1 μg FLAG–3C-expressing plasmid or empty FLAG vectors. At 24 hpt, the target proteins were analyzed by western blotting. (**C**) The cleavage of PABPC1 by 3C^pro^ in vitro was performed as the Materials and Methods section described. (**D**) HEK293T cells were transfected with 1.5 μg FLAG–3C expressing plasmid or 1.5 μg empty FLAG vector. At 24 hpt, the expression of 3C and PABPC1 was detected by immunofluorescence assay (IFA). Cells were double-immunostained for FLAG–3C (green) and PABPC1 (red); cellular nuclei were counterstained with 4’,6-diamidino-2-phenylindole (DAPI) (blue). (**E**) HEK293T cells cultured in 3.5-cm dishes were transfected with 1 μg FLAG–3C-expressing plasmids, 1 μg FLAG–3C mutant-expressing plasmids, 1 μg FLAG–PABPC1 plasmid, or 1 μg empty FLAG vector. At 24 hpt, the cells were analyzed by western blotting.

The 3C^pro^ of several picornaviruses has been identified to cleave host PABPC1 [26,27,28,29,30]. Therefore, we investigated the impact of SVV 3C^pro^ on the integrity of PABPC1. HEK293T cells were transfected with different doses of FLAG–3C-expressing plasmids or empty FLAG vectors. At 24 hpt, the cells were analyzed by western blotting. 3C^pro^ cleaved endogenous PABPC1 protein in a dose-dependent manner (Figure 2A).

To detect the N terminal cleaved band of PABPC1 induced by 3C^pro^, HEK293T cells were transfected with FLAG–PABPC1 plasmids along with FLAG–3C-expressing plasmids or empty FLAG vectors. At 24 hpt, the target proteins were analyzed by western blotting using an anti-FLAG antibody. The results were similar to SVV infection-induced cleavage of PABPC1, the overexpression of 3C^pro^ also induced the cleavage of exogenous PABPC1, generating approximately 50 kDa of N terminal cleaved band (Figure 2B).

To further confirm the impact of 3C^pro^ on exogenous PABPC1, the purified N terminal His-tagged PABPC1 protein and FLAG–3C protein were incubated in vitro. The PABPC1 protein was analyzed by western blotting using anti-His and anti-PABPC1 antibodies. The results indicated that 3C^pro^ can cleave PABPC1 in vitro (Figure 2C).

The subcellular colocalization of 3C^pro^ and PABPC1 was also examined by indirect immunofluorescence assay (IFA). The HEK293T cells were transfected with the plasmids expressing 3C^pro^. The distribution of 3C^pro^ and PABPC1 were detected. It showed that PABPC1 distributed predominantly in the cytoplasm with a diffused pattern, and we found that 3C^pro^ significantly induced the reduction in PABPC1 (Figure 2D).

The protease activity of 3C^pro^ plays an essential role in catalyzing the cleavage of host proteins [12,17]. To evaluate whether the 3C^pro^ protease activity was also responsible for this process, the plasmids expressing the 3C^pro^ with mutations at the critical protease sites were constructed, and the HEK293T cells were transfected with FLAG–3Cwt-expressing plasmids, FLAG–3C-mutant-expressing plasmids or empty FLAG vectors. At 24 hpt, the cells were collected and analyzed by western blotting. Abrogation of the protease activity of 3C^pro^ completely restored the integrity of PABPC1, suggesting that SVV 3C^pro^ cleaved endogenous PABPC1 depending on its protease activity (Figure 2E, left). To confirm the impact of protease activity of 3C^pro^ on exogenous PABPC1, HEK293T cells were transfected with FLAG–PABPC1 plasmid along with FLAG–3Cwt-expressing plasmid, FLAG–3C-mutant-expressing plasmids or empty FLAG vectors. At 24 hpt, the cells were analyzed by western blotting. It showed that SVV 3C^pro^ also cleaved exogenous PABPC1 through its protease activity (Figure 2E, right).

To further confirm the impact of 3C^pro^ protease activity on PABPC1 during SVV infection, we tried to rescue three SVV strains that contained the mutated sites at the protease activity of 3C^pro^ to abolish the protease activity of 3C^pro^: by introducing single-site mutations H48A and C160A, or doublesite mutations H48A–C160A, as described previously [12]. However, all three 3C^pro^ mutant strains could not be rescued successfully, which suggests that sites 48 and 160 of 3C^pro^ are essential for SVV replication.

Taken together, our results indicate that SVV 3C^pro^ induces cleavage of PABPC1, and that the protease activity of 3C^pro^ is essential for this cleavage. In addition, 3C^pro^ is essential for SVV replication.

### 2.3. Cleavage Sites of PABPC1

**Figure 3:**
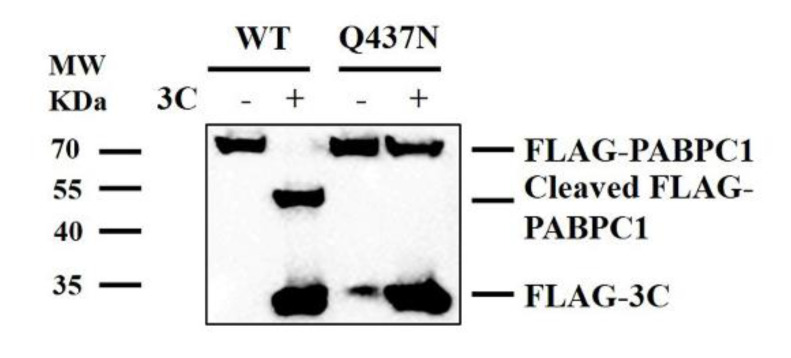
Cleavage sites of PABPC1. HEK293T cells were cultured in 3.5-cm dishes, and the monolayer cells were transfected with 1 μg FLAG–PABPC1 wild type (WT) plasmid and FLAG–PABPC1 Q437N plasmid along with 1 μg FLAG–3C-expressing plasmid or 1 μg empty FLAG vector. At 24 hpt, the cells were analyzed by western blotting.

Encephalomyocarditis virus (EMCV) 3C proteinase induced cleavage of PABPC1 at residue 437, generating approximately 50 KDa and 25 KDa cleaved bands [30]. To explore the cleavage sites of PABPC1 induced by SVV, we generated a FLAG–PABPC1 mutant (Q437N), as described previously [30]. HEK293T cells were transfected with a FLAG–PABPC1 wild type (WT) plasmid and FLAG–PABPC1 mutant (Q437N) plasmid, along with a FLAG–3C-expressing plasmid or empty FLAG vectors. At 24 hpt, the cells were analyzed by western blotting. 3C^pro^ cleaved WT PABPC1 protein, whereas it did not cleave PABPC1 mutant (Q437N) protein (Figure 3). The results show that residue 437 is important for the cleavage of PABPC1 induced by SVV 3C^pro^.

### 2.4. SVV 3C^pro^ Protease Activity Was Essential for Inhibition of the Protein Synthesis Rates

**Figure 4:**
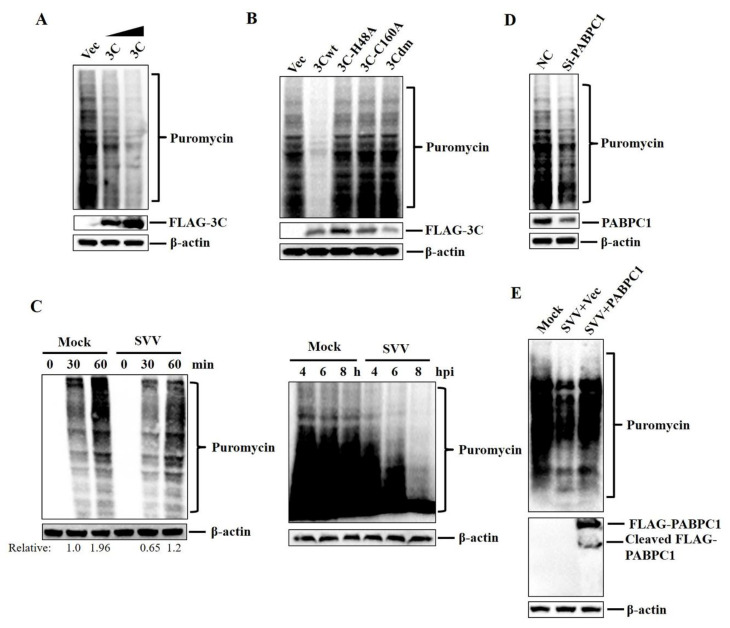
SVV 3C^pro^ protease activity was essential for inhibition of the protein synthesis rates. (**A**,**B**) HEK293T cells were cultured in 3.5-cm dishes, and the monolayer cells were transfected with 0, 0.5, and 1 μg FLAG–3C-expressing plasmids, 1 μg FLAG–3C mutant-expressing plasmids, or 1 μg empty FLAG vector. At 24 hpt, the cells were labeled with 10 µg mL^−1^ of puromycin for 1 h. Then, the cells were collected and analyzed by western blotting. (**C**) HEK293T cells cultured in 3.5-cm dishes were mock-infected or infected with SVV (MOI 1). At 4 hpi, the cells were labeled with 10 µg mL^−1^ of puromycin and then were collected at 0, 30, and 60 min after puromycin treatment (left). At 4, 6, and 8 hpi, the cells were labeled with 10 µg mL^−1^ of puromycin for 60 min (right). The cells were then analyzed by western blotting and quantified as described in “Materials and Methods” section. (**D**) HEK293T cells cultured in 3.5-cm dishes were transfected with 150 nM NC siRNA or PABPC1 siRNA. At 36 hpt, the cells were labeled with 10 µg mL^−1^ of puromycin for 1 h. The cells were then collected and analyzed by western blotting. (**E**) HEK293T cells cultured in 3.5-cm dishes were transfected with 1 μg FLAG–PABPC1 expressing plasmids or 1 μg empty FLAG vector. At 24 hpt, the cells were mock-infected or infected with SVV (MOI 1) for 5 h. Then, the cells were labeled with 10 µg mL^−1^ of puromycin for 1 h. The whole-cell lysates were analyzed by western blotting.

To confirm whether the cleavage of PABPC1 potentially affects the protein synthesis rates, HEK293T cells were transfected with FLAG–3Cwt-expressing plasmids, FLAG–3C-mutant-expressing plasmids or empty FLAG vectors. At 24 hpt, the cells were labeled with 10 µg ml^−1^ of puromycin [31]. Puromycin can be incorporated into the nascent polypeptide chain and prevents elongation, reflecting the protein synthesis rates [31]. The whole-cell lysates were analyzed by western blotting using an anti-puromycin antibody. SVV 3C^pro^ inhibited the protein synthesis rates in a dose-dependent manner (Figure 4A). However, 3C^pro^ mutants without protease activity failed to inhibit the protein synthesis rates (Figure 4B).

We also assessed the protein synthesis rates during SVV infection. HEK293T cells were mock-infected or infected with SVV (MOI 1), and the cells were labeled with 10 µg mL^−1^ of puromycin at the indicated time points. The whole-cell lysates were analyzed by western blotting. The protein synthesis rates were significantly higher in the mock-infected cells at 30 and 60 min after puromycin incubation compared with that in the SVV-infected cells (Figure 4C). In addition, SVV infection can inhibit the protein synthesis rates over time (Figure 4C).

We assessed the impact of PABPC1 on the protein synthesis. HEK293T cells were transfected with PABPC1 siRNA and negative control (NC) siRNA. At 36 hpt, the cells were labeled with 10 µg mL^−1^ of puromycin. The cells were then analyzed by western blotting. The protein synthesis rates were lower in the PABPC1 siRNA-transfected cells compared with that in the NC siRNA-transfected cells (Figure 4D), which suggested that PABPC1 also played important roles in cell translation process.

To further confirm the impact of PABPC1 on the protein synthesis rates, HEK293T cells that transfected with FLAG–PABPC1 expressing plasmids or empty FLAG vector were mock-infected or infected with SVV (MOI 1) for 5 h. Then, the cells were labeled with 10 µg mL^−1^ of puromycin for 1 h. The whole-cell lysates were analyzed by western blotting. The addition of PABPC1 effectively reversed SVV-induced blockage of the protein synthesis (Figure 4E). Taken together, our results indicate that SVV infection (or the overexpression of 3C^pro^) inhibits the protein synthesis rates by cleavage of PABPC1.

### 2.5. PABPC1 Inhibited SVV Replication during Viral Infection

**Figure 5:**
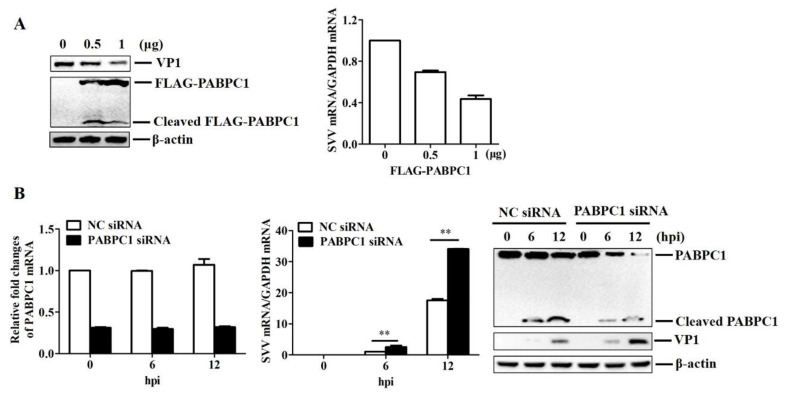
PABPC1 inhibited SVV replication during viral infection. (**A**) HEK293T cells cultured in 3.5-cm dishes were transfected with 0, 0.5, and 1 μg FLAG–PABPC1-expressing plasmids. At 24 hpt, the cells were infected with SVV (MOI 1) for 12 h. Expression of viral RNA was determined by qPCR; expression of PABPC1 and viral VP1 proteins was determined by western blotting. (**B**) HEK293T cells cultured in 3.5-cm dishes were transfected with 150 nM NC siRNA or PABPC1 siRNA for 36 h followed by infected with equal amounts of SVV (MOI 1) for 0, 6, and 12 h. The expression of PABPC1 mRNA and viral RNA was determined by qPCR; the expression of PABPC1 and viral VP1 proteins was determined by western blotting. ** *p*-value <0.01.

The impact of PABPC1 on SVV replication remains unclear. To address this question, we assessed SVV yields in HEK293T cells transfected with different doses of FLAG–PABPC1-expressing plasmids. At 24 hpt, the cells were infected with SVV (MOI 1) for 12 h. Viral RNA and VP1 protein levels were compared. The overexpression of PABPC1 significantly inhibited SVV RNA and protein levels in a dose-dependent manner (Figure 5A).

We also assessed SVV yields in PABPC1-down-regulated cells. HEK293T cells were transfected with PABPC1 siRNA and NC siRNA. At 36 hpt, the cells were infected with SVV (MOI 1). Viral RNA, VP1 protein, and host PABPC1 protein levels were compared at the indicated time points (0, 6, and 12 h) after infection. This showed that SVV replication was significantly enhanced in the PABPC1 siRNA cells compared with that in the NC siRNA cells (Figure 5B). Taken together, our results indicate the important antiviral role of PABPC1 against SVV replication.

## 3. Discussion

It is well known that the cell translation process is essential for cell survival and antiviral function. Many virus replications rely on cell translation factors [20,28,32,33]. However, some viruses have evolved some strategies to cleave or reduce cellular eIFs [26,34,35] in order to shut off host protein synthesis and promote virus replication. In this study, for the first time, we investigated the impact of SVV on PABPC1 protein. Our results showed that SVV infection resulted in the cleavage of PABPC1.

The impact of other viruses on PABPC1 has also been reported. For example, rotavirus nonstructural protein 3 (NSP3) interacts with eIF4G and displaces the PABP protein from eIF4F [35]; calicivirus 3C-Like proteinase inhibits cell translation by cleavage of PABP [36]; hepatitis A virus (HAV) 3C proteinase cleaves PABP in vitro and vivo [37]; coxsackievirus B3 2A^pro^ can cleave PABP [23]; EMCV 3C proteinase induces cleavage of PABP [30]; poliovirus 3C protease cleaves PABP and eIF4G to inhibit host cell translation [24,27]; duck hepatitis A virus (DHAV) 3C protease cleaves PABP [28]; and FMDV infection induces proteolytic of PABP [26,38]. The protease activity of 3C^pro^ plays an important role in this process. Here, our results showed that SVV 3C^pro^ induced cleavage of PABPC1. A detailed data analysis indicated that cleavage of PABPC1 was dependent on the protease activity of SVV 3C^pro^. Together, these results showed that PABPC1 can be cleaved by a number of viral proteinases of different viruses.

The carboxyl terminal regions (residues 413, 437, and 537) of PABPC1 were the target sites invloved in cleavage [24,29,37,39]. Here, PABPC1 was cleaved into two fragments (~50 kDa and ~25 kDa), and residue 437Q was identified as the cleavage site recognized by SVV, in accordance with that recognized by EMCV 3C^pro^ [30]. These results show that the 3C^pro^ of various picornaviruses may cleave PABPC1 by similar mechanisms.

The impact of PABPC1 on viral replication has been reported. Some viruses utilize host translation factors to promote their own replication; however, there are also some viruses that do not use host translation factors for their own replication [40,41]. For instance, PABPC1 promotes cytomegalovirus and DHAV replication [28,41]; cleavage of PABPC1 stimulates EMCV replication [30]; PABPC1 is a positive modulator of dengue virus replication [25] and PABP1 inhibits HAV replication [37]. In the present study, our results indicated that PABPC1 restricted SVV replication. The results reveal the diverse functions of PABPC1 during different viruses infection.

Cellular mRNAs contain a 5′ 7-methylguanosine (m7G) cap structure, which is essential for binding to the eIF4F complex, including the eIF4E, eIF4A, and eIF4G proteins [32]. However, picornaviruses use a cap-independent mechanism that binds to initiation factors by its own internal ribosome entry site (IRES) [42,43]. Studies have indicated that many picornaviruses reduce host antiviral response and competition between the viral and cellular RNAs for translation components by inhibiting host cell translation [32]. Here, for the first time, we showed that SVV inhibits host cell translation by cleaving PABPC1, resulting in a decreasing host antiviral response and promoting the translation of viral RNA preferentially. SVV enters into host cells through anthrax toxin receptor 1 [44]. After that, SVV induces the cleavage of PABPC1 to promote viral replication, resulting in severe infection and specific clinical symptoms, which provides a novel pathogenesis mediated by SVV. A better understanding of this viral pathogenesis could lead to the development of vaccines and therapeutic interventions.

In conclusion, for the first time, we showed that SVV infection cleaves PABPC1, and 3C^pro^ protease activity is essential for this process. The cleavage of PABPC1 results in the inhibition of the protein synthesis rates. Our results also indicate the important antiviral role of PABPC1 on SVV replication.

## 4. Materials and Methods

### 4.1. Cells, Viruses and Infection

HEK293T and PK-15 cells prepared in our laboratory were cultured in Dulbecco’s modified Eagle medium (Thermo Fisher Scientific, Waltham, MA, USA) supplemented with 10% heat-inactivated fetal bovine serum (FBS) (Thermo Fisher Scientific) and maintained at 37 °C (5% CO_2_). The SVV strain isolated in our laboratory was used for viral challenge [2].

### 4.2. Plasmids and Antibodies

The cDNA of human PABPC1 was amplified from HEK293T cells and cloned into pcDNA3.1(+)-FLAG vector (Invitrogen, Carlsbad, CA, USA) to yield the N terminal FLAG-tagged expression construct (FLAG–PABPC1). The FLAG–PABPC1 mutant (Q437N) was generated using a specific primer, as described previously [30]. The FLAG-tagged SVV 3C^pro^ construct and 3C^pro^ mutants (H48A, C160A, H48A-C160A) without protease activity were prepared in our laboratory [17]. All constructed plasmids were analyzed and verified by DNA sequencing.

The commercial antibodies used in this study included: anti-FLAG monoclonal antibody (Santa Cruz Biotechnology, Santa Cruz, CA, USA), anti-FLAG polyclonal antibody (Sigma–Aldrich, St. Louis, MO, USA)anti-PABPC1 polyclonal antibody (Abcam, Cambridge, MA, USA), anti-puromycin monoclonal antibody (Merck & Co., Kenilworth, NJ, USA), and anti-β-actin monoclonal antibody (Santa Cruz Biotechnology). Anti-VP1 polyclonal antibody was prepared in our laboratory [17].

### 4.3. Western Blotting

For western blotting anslysis, the target proteins were analyzed by 10% sodium dodecyl sulfate-polyacrylamide gel electrophoresis (SDS-PAGE) and transferred onto an Immobilon-P membrane (Millipore, Bedford, MA, USA). Then, the membrane was blocked with 5% skimmed milk for 2 h at room temperature, and incubated with primary and secondary antibodies at 4 °C, as described previously [45,46]. The antibody–antigen complexes were visualized by chemiluminescence detection reagents (Thermo Fisher Scientific). The change in the abundance of puromycin was determined by densitometric analysis using ImageJ Software (version 1.39, NIH, Bethesda, MD, USA) and normalized to β-actin.

### 4.4. Indirect Immunofluorescence Microscopy

HEK293T cells cultured in Nunc™ glass bottom dishes were transfected with empty FLAG vector or FLAG–3C plasmids. At 24 hpt, the cells were fixed with formaldehyde for 12 h at −4 °C. The cells were incubated with anti-FLAG and anti-PABPC1 primary antibodies overnight at 4 °C. The fluorochrome-conjugated secondary antibodies were then used in a reacttion with the primary antibodies in the dark for 6 h at 4 °C. After that, the cells were stained with 4’, 6-diamidino-2-phenylindole (DAPI) for 10 min to reveal the nuclei. The fluorescence was visualized using a Nikon Eclipse 80i fluorescence microscope.

### 4.5. Expression and Purification of Recombinant PABPC1 and SVV 3C^pro^ Proteins

The prokaryotic expression and purification of N-terminal His-tagged PABPC1 protein was purchased from Zoonbio Biotechnology company, Nanjing, China.

HEK293T cells were cultured in 10-cm dishes, and the monolayer cells were transfected with empty FLAG vector or FLAG–3C plasmids. The collected cells were lysed by lysis buffer, and the FLAG–3C protein was purified with FLAG affinity resin (Thermo Fisher Scientific) according to the manufacturer’s protocol.

### 4.6. In Vitro Cleavage Reaction

The in vitro cleavage reaction was performed as described previously [28,47]. Briefly, 3 μg of purified His-tagged PABPC1 protein (final concentration, 1 mg/mL) and 300 ng of purified FLAG–3C protein (final concentration, 50 μg/mL) were incubated for 12 h at 16 °C using cleavage buffer (20 mM Tris-HCl (pH 7.0), 5 mM DTT, 150 mM NaCl, 10% glycerol). Then, the reaction was stopped with the 4*SDS-PAGE sample buffer. The target proteins were analysed by western blotting with anti-PABPC1 and anti-His antibodies.

### 4.7. Knockdown of PABPC1 Using siRNA

The siRNAs used in the RNAi assay were synthesized by Gene Pharma (Shanghai, China). The knockdown of endogenous PABPC1 in HEK293T cells was performed by the transfection of PABPC1 siRNA using Lipofectamine 2000 (Invitrogen). The siRNA was used as a negative control (NC). The human PABPC1 siRNA sequence is F: GACGAUUUAAGUCUCGUAATT, R: UUACGAGACUUAAAUCGUCTT.

### 4.8. RNA Extraction and qPCR

Total RNA was extracted from the samples with TRIzol^®^ reagent (Thermo Fisher Scientific) according to the manufacturer’s protocol. The cDNA was prepared as described previously [48]. SYBR Premix Ex Taq reagents (TaKaRa, Dalian, China) were used for the qPCR assay. The housekeeping gene glyceraldehyde-3-phosphate dehydrogenase (GAPDH) was used as an internal control. Relative mRNA expression was calculated with the comparative cycle threshold (CT) (2^−ΔΔCT^) method [49]. The qPCR primers used in this study are listed in Table 1.

### 4.9. Puromycin Labeling

The puromycin labeling assay that was used for monitoring host protein synthesis was performed as described previously [31,50]. Briefly, HEK293T cells were transfected with various plasmids, PABPC1 siRNA, mock-infected or infected with SVV. At the indicated time points, the cells were labeled with 10 µg mL^−1^ of puromycin (Selleckchem, Houston, TX, USA). The cells were then analyzed by western blotting using anti-puromycin antibody.

### 4.10. Statistical Analysis

Statistical analysis was performed using SPSS Statistics for Windows, Version 17.0 (SPSS Inc., Chicago, IL, USA). Student’s *t* test was used for the comparison of three independent experiments. A * *p*-value <0.05 was considered significant; A ** *p*-value <0.01 was considered highly significant.

## Figures and Tables

**Table 1:** qPCR primers used in this study.

Primers	Sequences (5′-3′)	Target Gene
SVV-F	AGAATTTGGAAGCCATGCTCT	SVV gene
SVV-R	GAGCCAACATAGARACAGATTGC	
PABPC1-F	GGTTATGATGGAGGGTGGTCGC	human PABPC1 gene
PABPC1-R	GGGGTTGATTACAGGGTTGGGA	
GAPDH-F	CGGGAAGCTTGTGATCAATGG	human GAPDH gene
GAPDH-R	GGCAGTGATGGCATGGACTG	
